# NeuroChaT: A toolbox to analyse the dynamics of neuronal encoding in freely-behaving rodents *in vivo*

**DOI:** 10.12688/wellcomeopenres.15533.1

**Published:** 2019-12-09

**Authors:** Md Nurul Islam, Seán K. Martin, John P. Aggleton, Shane M. O’Mara

**Affiliations:** 1 Institute of Neuroscience, Trinity College Dublin, Dublin 2, Ireland; 2 School of Psychology, Cardiff University, Cardiff, UK

**Keywords:** Cognitive map, data analysis, spatial coding, single unit, open software, electrophysiology

## Abstract

There is a dearth of freely-available, standardised open source analysis tools available for the analysis of neuronal signals recorded *in vivo *in the freely-behaving animal. In response, we have developed a freely-available, open-source toolbox, NeuroChaT ( Neuron Characterisation Toolbox), specifically addressing this lacuna. Although we have particularly emphasised single unit analyses for spatial coding, NeuroChaT also characterises rhythmic properties of units and their dynamics associated with local field potential signals. NeuroChaT was developed using Python and facilitates a complete pipeline from automation of analysis to producing and managing publication-quality figures. Additionally, we have adopted a platform-independent format (Hierarchical Data Format version 5) for storing recorded and analysed data. By providing an easy-to-use software package, we aim to simplify the adoption of standardised analyses for behavioural neurophysiology and facilitate open data sharing and collaboration between laboratories.

## Introduction

Where and how spatial information is represented in the brain has been of great scientific interest since O’Keefe and Dostrovsky ^1^ first described the spatially-receptive fields of hippocampal neurons (since named ‘place cells’). Subsequently, many spatially-responsive cell types have been described, including head direction cells ^2, 3^, grid cells (neurons with multiple receptive fields arranged in a triangular grid) ^4, 5^, as well as boundary cells and object cells (neurons that respond to objects placed in the environment) ^6, 7^. Moreover, neurons tuned to non-spatial, natural stimuli (e.g. speed cells), have also been described, and are likely to contribute to the dynamic representations of ‘self-location’, such as for path integration ^8, 9^.

Standardised methods have evolved for studying the spatial selectivity of neurons in the freely-behaving animal. Briefly, rats (or mice) are surgically implanted with recording electrodes targeted at a particular brain region or regions. After post-surgery recovery, the freely-moving rat traverses mazes or open fields (often in search of food). The experimental apparatus may be shielded from the larger laboratory by curtains, to control the local cue set. This cue set may be manipulated with, for example, cue rotations or selective cue deletions. Neuronal activity (action potentials, or ‘spikes’) is recorded, amplified, time-stamped and correlated with the moment-to-moment position of the rat. These correlations are used to generate colour-coded contour maps representing the density of spike firing at all points occupied by the rat. Under these conditions, many hippocampal neurons fire in a locally defined area of the maze (usually no more than a few percent of the total maze area) and remain silent or fire at low rates (<1 Hz) in other areas of the maze ^9^.

Modern recording techniques may use multiple recording fine-wire electrodes or electrodes based on printed circuit technology ^10^. These approaches generate vast amounts of data, particularly if acquired over long duration recording sessions. Moreover, advances in the design of recording electrodes have increased the number of recording sites ^10, 11^, increasing data volumes ^12, 13^. Analysing such large data sets involves:

1. Identifying the activity of single neurons from the noisy recorded data, known as spike sorting ^14^.
2. Analysing relationships between spatial and non-spatial variables and verifying correlations.
3. Assessing individual neurons and computing inferential statistics to describe local populations.

There are some open-source software packages for studying the neural codes of single neurons, multiple neurons, and local field potentials ^15, 16^. Many individual laboratories use custom-written software, but there is no software package widely available implementing standardised algorithms for spatial and non-spatial neuronal coding within one working environment, thereby limiting wider adoption of *in vivo* electrophysiological recording methods. Nor is there a widely- and freely-available toolbox to analyse neuronal encoding of spatial and non-spatial information that also incorporates batch processing of substantial amounts of data. Finally, available packages do not often easily facilitate quick implementation and integration of new techniques along with established ones given the challenges associated with the evolution of new technology.

To address this important lacuna, we have developed a toolbox, NeuroChaT (Neuron Characterisation Toolbox), a graphical user interface (GUI)-based open-source software that brings together peer-reviewed analysis methods in a unified framework for greater accessibility and to provide an easier implementation of analyses. We have adopted the widely-used platform-independent Hierarchical Data Format version 5 (HDF5) for storing recorded and analysed data, which is compatible with most common programming languages. NeuroChaT provides a systematic approach for analysing large numbers of neurons and managing the graphical and parametric outputs. NeuroChaT is freely available from GitHub ( https://github.com/shanemomara/omaraneurolab) under the GNU General Public License (v3.0) for non-commercial use and open source development. Sample data, a GUI user tutorial and extensive application programming interface (API) documentation are also provided on the project website. We hope NeuroChaT will enable standardisation of analyses and assist in developing novel algorithms and experimental designs through its ease of analysis based on a widely-used and standardised data format.

## Methods

### Analysis methods

**Figure 1.**
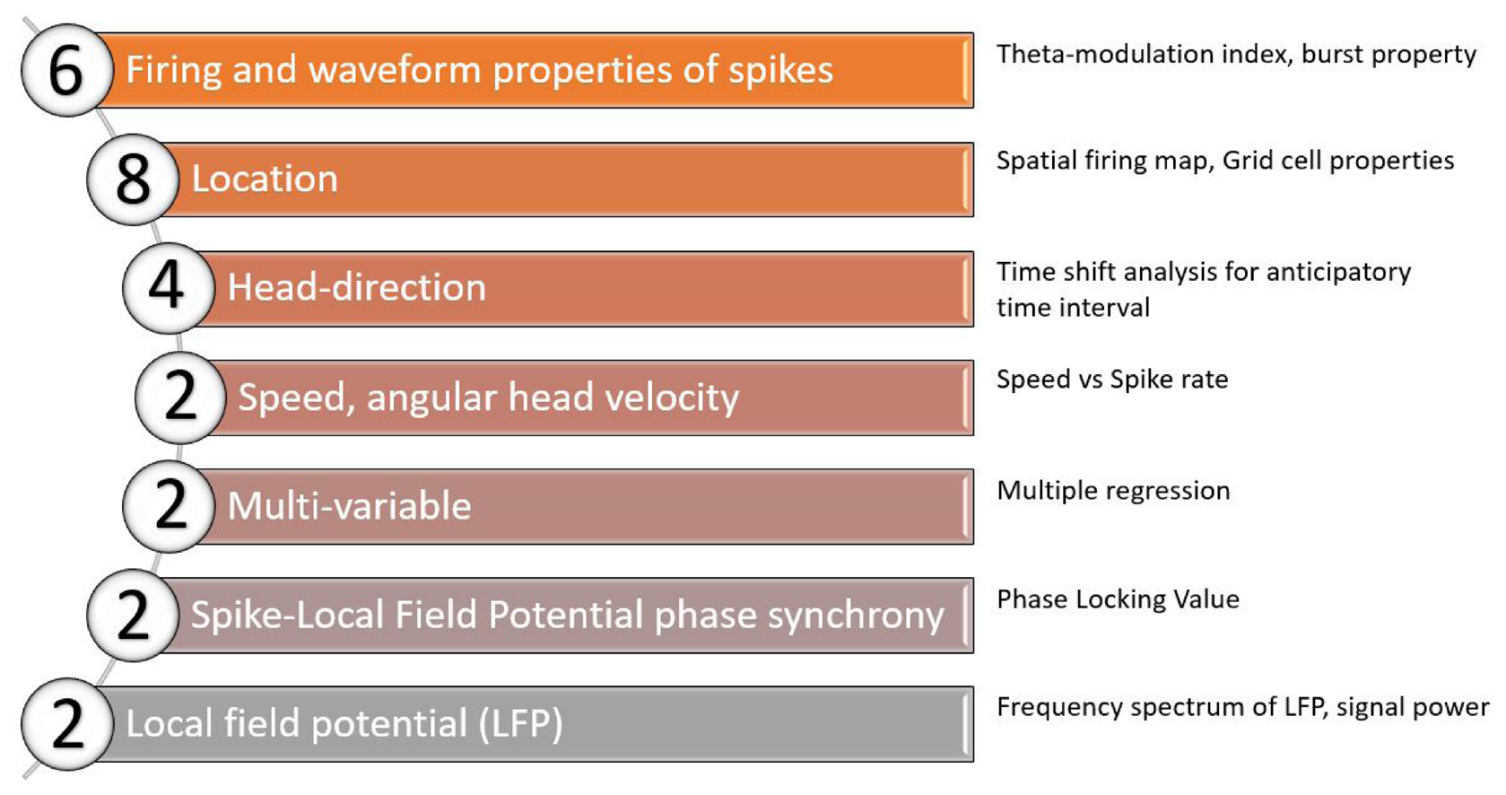
The number of methods available in NeuroChaT for each category of analysis.

**Figure 2.**
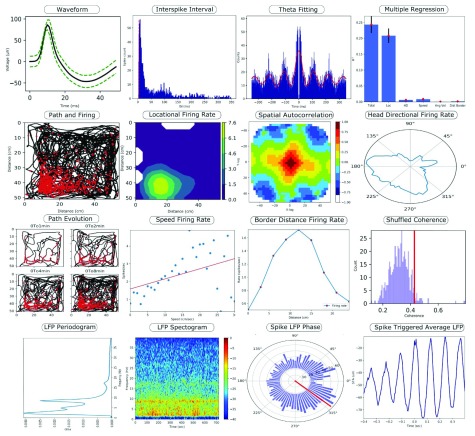
Example plots to demonstrate the graphical output from a subset of the analyses available in NeuroChaT. A short description of each plot follows, going from top left to bottom right and moving along rows. (1) The mean waveform of a single unit (black) and the standard deviation of the waveform (green). (2) The histogram of the interspike interval of a unit, with the red dotted line showing the refractory period. (3) A wave fitted to the autocorrelation of the interspike interval at theta frequency (8 Hz). (4) The predictive power of location, head direction, speed, angular velocity, and border distance for the firing rate. (5) The path of the rodent in a square arena (black) and firing (red). (6) The locational firing rate information modulated by dwell time in the arena, with green indicating high firing rates. (7) The spatial autocorrelation of the locational firing rate map, with red indicating high spatial autocorrelation. (8) A polar plot showing the firing rate modulated by head direction. (9) The path of the animal in the arena and firing activity over time. (10) A scatter plot of speed against the firing rate, with the red line showing a line of best fit. (11) A line plot comparing the border distance to the firing rate. (12) A histogram of spatial coherence values for 500 shuffled spike trains, with the red line indicating the 95 *th* percentile value. (13) The power in the local field potential (LFP) signal at different frequencies. (14) The power of the LFP signal at different frequencies over time, with red indicating high power values. (15) A polar plot of the LFP phase value at each spike time, with the red line indicating the mean phase. (16) The average LFP signal around the time of a spike occurrence.

NeuroChaT consists of multiple analysis methods that produce graphical figures and numerical results based on the normative neuronal rate coding scheme, where changes in firing rate represent responses to a stimulus or stimuli. The methods that are available in NeuroChaT are enumerated in Figure 1 and some example graphical outputs are shown in Figure 2.

NeuroChaT provides six analysis methods for assessing the waveform and firing properties of single units. Waveform properties measure characteristics such as the mean wave amplitude and width on each tetrode in a recording. The inter-spike interval (ISI), ISI autocorrelation, and cell bursting properties are calculated from the spike train of the single unit. In addition, a theta-modulated cell index and theta-skipping cell index for the single unit are both calculated by fitting an oscillating curve to the ISI autocorrelation histogram.

NeuroChaT offers eight spatial locational analyses. The spatial path of the subject and the spike train are used to produce a locational firing rate map. From the firing rate map, place field, grid cell, border cell, and gradient cell analyses are available. The place field is determined by finding the connected area of activity in the arena with the highest firing rate. Grid cell analysis involves calculating the spatial autocorrelation of the firing rate map and assessing the shape formed by the peaks in autocorrelation. For border cell analyses, a border of the arena is estimated from the path the animal traversed and the firing rate is compared to the distance from the border. Gradient cell analysis begins similarly to border cell analysis and then fits a Gompertz function (a monotonically increasing function that exhibits a slow growth rate at the border and the centre of the arena) to the relationship between firing rate and the distance from the border.

The following three methods are shared between spatial locational and head directional analyses. Time-lapse analyses examine the evolution of the firing rate over time to determine if spatial tuning occurs during the animal’s exploration of the environment. Shuffling tests randomly distribute the original spikes along the path of the animal to investigate whether the effect of a spatial variable on the firing rate of a unit has occurred by chance. Time-shift analyses gradually move the whole spike train of a unit forwards and backwards in time to test if there is a corresponding gradual change in the coding specificity, indicating a systematic variation in the firing rate and providing timing information of the spatial cells ^17^. Skaggs information content ^18^ is available in NeuroChaT for any spatial variable and is appropriate to use in combination with these spike time-altering analyses. Furthermore, for locational analyses, these methods can be used in combination with coherency and sparsity measures, which assess the spatial quality of a single unit. For head directional analyses, these methods can be used with the Rayleigh Z-score and the concentration parameter for the von Mises distribution, which assess the uniformity of the head-direction firing rate.

Head directional firing rate analyses are also available. These compare the spike train information to the head direction of the animal and can be computed for different angular velocities, such as when the animal is turning clockwise or counterclockwise. To round off NeuroChaT’s single variable spatial analysis toolkit, there are two analyses methods related to speed and angular velocity. In these, the spike rate is linearly correlated to the speed of the animal and the angular velocity of the animal’s head in both the clockwise and counter-clockwise directions.

There are two multi-variable spatial analyses in NeuroChaT. The first involves building a multi-variable linear regression model to predict the firing rate of a single unit. The location, head direction, speed, angular velocity, and distance to the border are the five predictor variables used to estimate the firing rate of the unit at multiple binned points in time. The predictive power of these variables is indicative of the spatial tuning of the single unit. The second analysis compares the observed firing rate related to an independent variable (speed, angular velocity, distance to the border, or head direction) to an estimated firing rate. The estimated firing rate is formed solely from the binned locational firing rate map and the value of the independent variable in each locational bin. In this way, it can be determined if modulation of the firing rate by an independent variable is a real effect, or if it is attributable to an inhomogeneous sampling of the independent variable.

There are two analysis methods available in NeuroChaT to analyse the raw local field potential (LFP) signal. The first involves computing the time-resolved frequency spectrum of the LFP. The second involves computing the average power in the LFP over the duration of the recording in the different frequency bands, such as the Theta band, using Welch’s periodogram. When considering the LFP in relation to the spiking information, two analyses are available. In the first analysis, the spike-triggered average LFP signal, the phase-locking value, and the spike-field coherence measures are obtained to assess the phase-locking of a unit to the LFP signals. In the second analysis, the distribution of the phase in the LFP at which spikes occur is formed by using the Hilbert Transform of the band-pass filtered LFP signal.

In addition to the analyses listed in Figure 1, two uncategorised methods are available in NeuroChaT. NeuroChaT can compute the Hellinger distance and the Bhattacharyya coefficient between spike clusters to evaluate the separation of unit clusters on a tetrode or to compare the similarity of a cluster across recordings. The latter can be used to help identify if the same cell is present in multiple recordings. To aid analysing substantial amounts of data, NeuroChaT can produce a summary png plot of the spatial information on each tetrode in multiple recordings. For Axona data, this can recursively search directories and produce a summary for any tetrode file with sorted spikes and is readily extendable to other formats.

### Implementation

**Figure 3.**
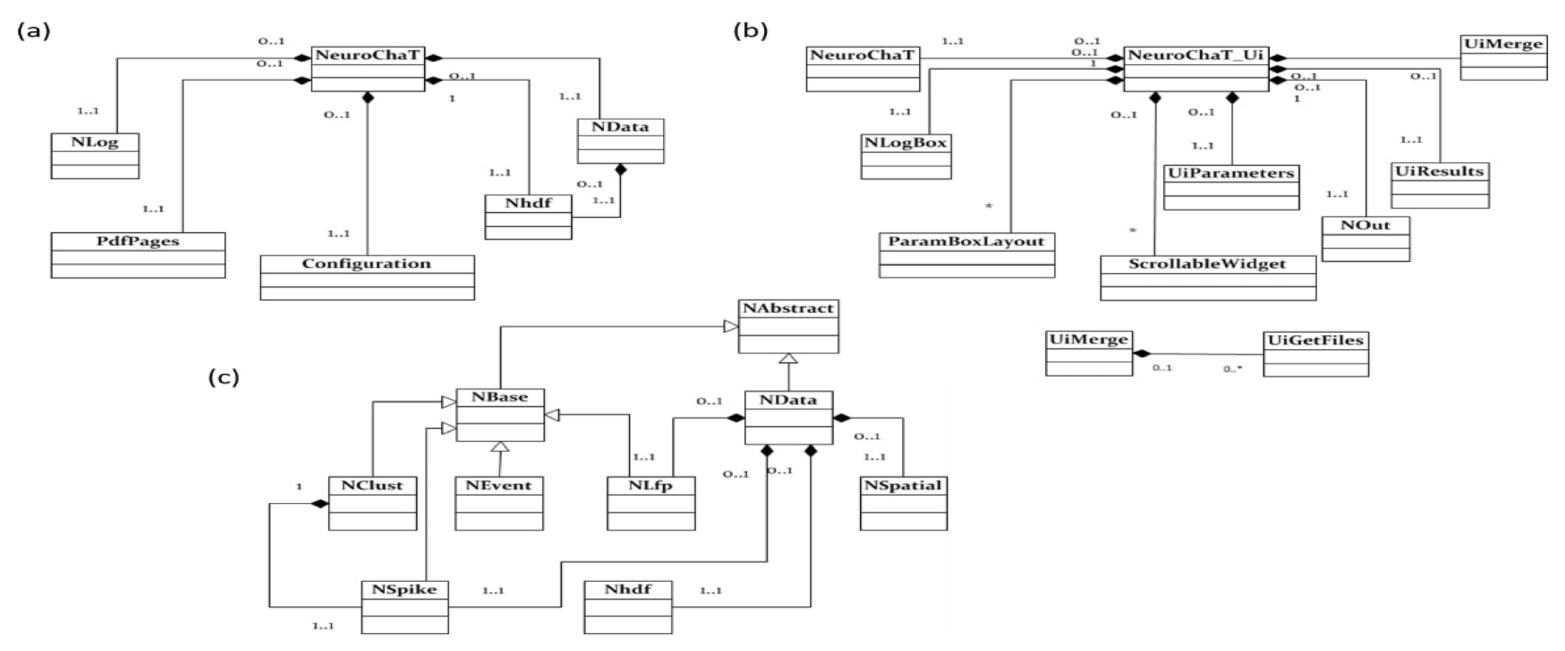
Class diagrams showing the relationships between the classes in NeuroChaT. Although each class contains several member attributes and local variables, these are not represented to keep the diagrams compact. The regular white arrows indicate class inheritance while the black diamond arrows indicate object composition. The numbers along an arrow specify the allowable number of instances in the relationship. For example, in the composition between NData and Nhdf, 1..1 indicates that an NData object has exactly one Nhdf object, while 0..1 indicates that an Nhdf object belongs to at most one NData object.

NeuroChaT uses object-oriented programming (OOP), using the freely-available open-source programming language, Python. In OOP, classes are programming elements that work as a placeholder for data and functions an object can perform, providing encapsulation of its attributes and actions. The relationships between the classes are shown in Figure 3 using class diagrams. The classes were designed to encapsulate one aspect of the software. For example, the NeuroChaT UI class manages the GUI and corresponding interactions between the graphical elements with the underlying code and data containers.

***NeuroChaT class (NeuroChaT).*** The NeuroChaT class takes information from GUI, determines what analysis or action to perform, and dictates to other connected classes to act accordingly. NData is a façade data structure composed of data classes and governs information flow between the other data classes, namely NSpike, NSpatial, NLfp and Nhdf. Data classes, like NSpike and NSpatial, are placeholders for spiking activity of neurons and the spatial position of the animal, respectively. NeuroChaT passes the relevant parameters to NData, and asks permission to perform the analyses, on a cell-by-cell basis, based on the user input in the specification phase.

***User interface class (NeuroChaT_Ui).*** The NeuroChaT user interface class is the graphical component of the NeuroChaT software. It provides the interface for users to specify the analyses they want to perform, select the data and parameters for those analyses, and, finally, the graphical file format to store the results in. This is a simple-to-use, tick-box interface with features that enable settings and information to be forwarded to the NeuroChaT object. Its composing objects are all graphical elements, except the NeuroChaT object. Although built in a composite structure, this class is static, in the sense that its components cannot be altered dynamically using commands outside of the class itself. Therefore, the coupling between these classes to others is considered tight, and any changes required must involve changing the code file where the class is defined.

***Neuronal spiking information class (NSpike).*** The NSpike class is the container for neuronal spiking activities. It decodes the files that record the waveforms and timestamps of the spikes from a proprietary format and stores these in a Neurodata Without Borders (NWB) format. It also contains analyses involving spiking activity of the single-units, i.e. inter-spike interval, assessing rhythmicity etc., along with implementing the decoders for the copyrighted data formats. If the recording undergoes spike-sorting, this class also provides the information about which spike-waveform belongs to which putative neuron.

***Neuronal local field potential class (NLfp).*** The NLfp class is the container for recorded LFP activities. The timestamps and the amplitude of the LFP information are stored in the instance of this class along with other recording information, i.e. LFP channel number or the bandwidth of the filter that was used to extract the LFP signal from the recorded data. The analyses that are implemented in the class are frequency spectrum of the LFP signal, LFP phase distribution, phase locking and SFC of an event-timestamp train as that of a single-unit, event-triggered average LFP signal etc.

***Spatial information class (NSpatial).*** The NSpatial class contains methods for analysing the spatial correlation of the single units. The only single unit information required for this class is the timing of the activity. This is passed directly as an input to its methods (API use guide) or through NData. When used with the NData class, it receives the information through that class instead of coupling directly to the NSpike data. This creates a layer of independence between the data classes and reduces the effort required to couple them.

***Neuronal spike sorting class (NClust).*** The NClust class provides the waveform features, unit spiking activity, and measures of cluster separation for quality assessment of spike-sorting and measuring the cluster similarity with a unit in another NClust object. The class delegates the handling of the file containing the neuronal spike information to the NSpike object that is an attribute of the NClust instance.

***Neuronal Hierarchical Data Format class (Nhdf).*** NData also contains an Nhdf data object to provide read/write access of HDF5 files containing spatial or neural data within the class without decoding the proprietary file formats every time the data is loaded. As the HDF5 file contains all the data, it makes storage more manageable through a readable format. Nhdf contains methods to read and write what is called groups and datasets in HDF5 file format. It also contains methods that are specific to storing individual NSpike, NLfp, and NSpatial data to their common HDF5 container for a recording session. NeuroChaT creates one such file for each recording session, not for individual units or electrodes.

***Neuro-data class (NData).*** The NData object, as shown in Figure 3, comprises data objects of different kinds, and is built upon the composite structural object pattern ^19^. This type of design pattern used in NeuroChaT creates a modular structure and allows the objects to alter dynamically without intense refactoring of the code. In NeuroChaT, NAbstract and NBase form the parent classes with basic and common methods and attributes across different data types. Each data class representing the neural data (NSpike, NClust, NLfp), along with the event class NEvent, inherits NBase, where NBase itself inherits NAbstract and extends its capabilities. The NSpatial class inherits the NAbstract class. The NData class gets one instance or object for each NSpatial, NSpike and NLfp class as its attribute. The rationale behind this design is to provide an encapsulation of the interaction among the behavioural and neural data types, i.e. how the peers like NSpatial and NSpike would know each other. Either they will need to have a reference to each other, which increases their coupling, or they need to be cooperated using another object, which, in our design, is the NData object.

A similar design principle is also followed in other composite classes of NeuroChaT. The getter and setter methods of the composite class instance then allow dynamically changing the objects or retrieving it. For example, the spatial data does not need to be changed for a single recording while analysing for multiple single units recorded in the same session. Therefore, the NSpatial object remains the same, but the data in the NSpike object changes with changing the units. Now, creating one instance of NData for every pair of spatial and single unit data is not very memory efficient. Instead, we can replace the data in the NSpike by reloading the spike file while it is still a member of the NData object and optimise the reuse of data objects, save memory, and increase the performance of the software.

***Experimental event class (NEvent).*** NEvent class implements event-related data management and basic analyses, i.e. peri-stimulus time histogram (PSTH) and analyses pertaining to locking of the LFP signals to the event(s). It also delegates the analyses to the relevant NSpike or NLfp objects. For example, if the PSTH is to be obtained from a spike-train, the NEvent object recruits the relevant NSpike and uses its function computes the same analysis.

***Visualisation and export.*** NeuroChaT uses a custom python module ‘nc_plot’ to plot the graphical outcomes of the analyses, then stores the parametric results in a tabular format and converts the data into the standardised HDF5 files using the Nhdf() object. The user can perform statistical analyses on the parametric results if required: this is the Inference phase of the data analysis workflow using NeuroChaT. The specifications of the data, analyses, and input parameters can be saved for future use in an ‘ncfg’ (NeuroChaT configuration) file. This file is in YAML format, a human-readable data-serialisation format commonly used for configuration files.

***Utility classes.*** In addition to the primary classes already described, NeuroChaT also provides classes that provide essential utility functionality. NLogBox is an editable graphical widget that is subclassed from QTextEdit of the QtWidgets of PyQt5 to format the logged messages into HTML format. ParamBoxLayout is derived from QVBoxLayout and is used for arranging the parameter definition in a vertical layout in the Settings menu of the interface. ScrollableWidget provides a container of listed items so the user can scroll through the items if the list takes more space than the widget they are located in. UiParameters define and add the graphical elements to the interface. NOut replaces the standard output texts of Python or IPython (print command) into texts that are received by the logger of the system. UiResults is a sub-class of QDialog of QtWidget that displays the results of the analysis in a tabular format along with an option to export them in an Excel file. UiMerge is a graphical window that asks the user to select a list of pdf filenames to merge them into one file or to transfer to a single folder. The user can also select the pdf files manually using an interactive window built in UiGetFiles class.

### Operation

NeuroChaT has been tested to run on Windows 7, Windows 10, and Ubuntu 18.04. NeuroChaT requires 100MB of system storage to perform a full install, including Python and Python package dependencies. There are no system requirements to run NeuroChaT, but at least 8GB of RAM is recommended.

**Figure 4.**
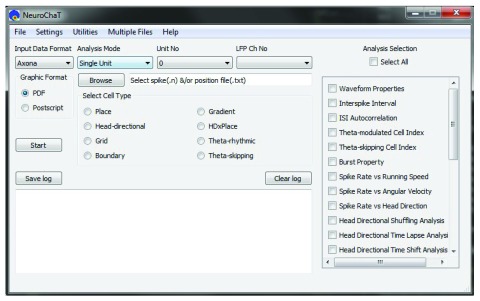
The graphical user interface to NeuroChaT.

**Figure 5.**
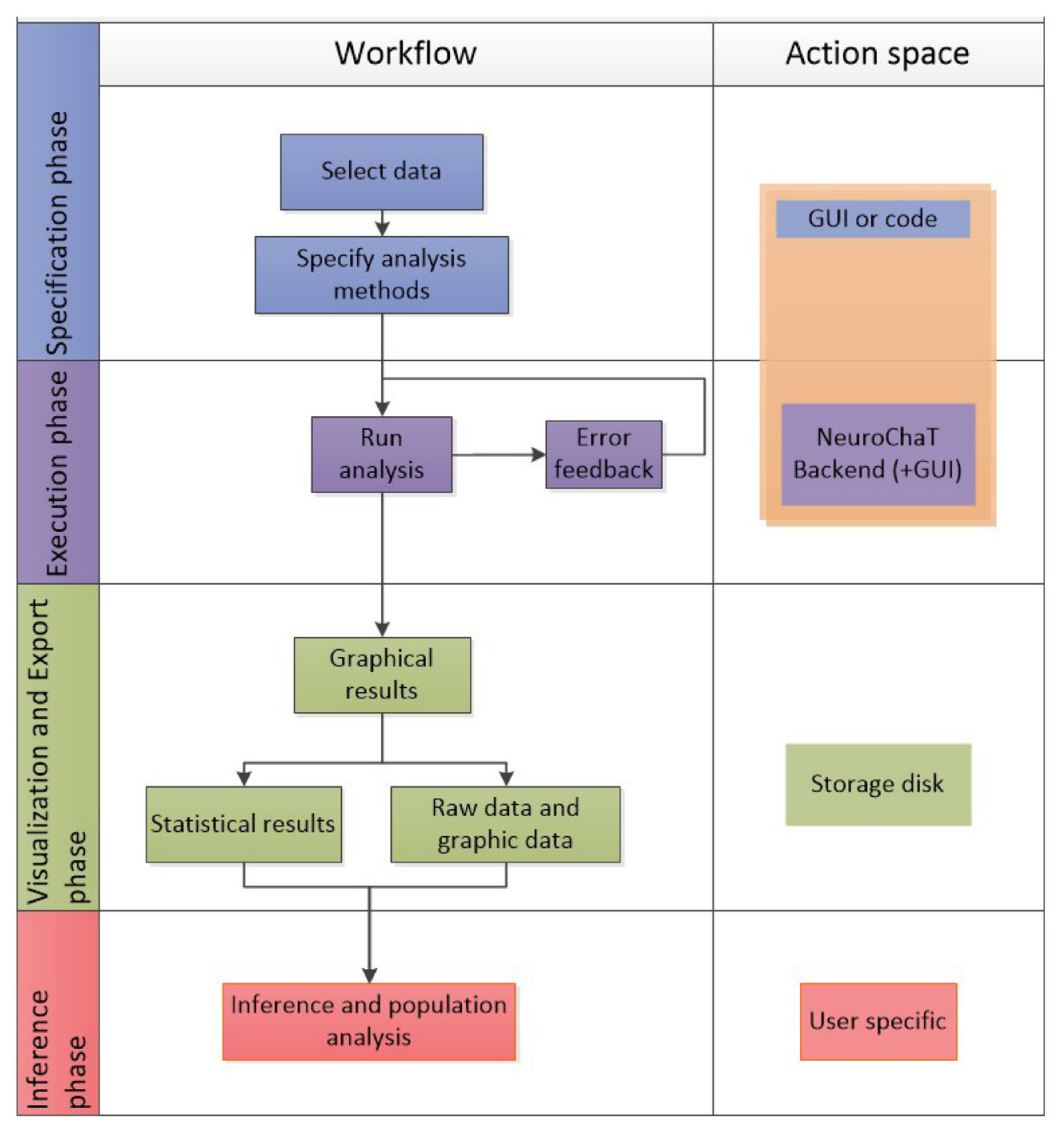
Linear workflow for using NeuroChaT.

The NeuroChaT graphical user interface (GUI) is shown in Figure 4. The linear workflow for using NeuroChaT is shown in Figure 5. Initial analysis specification starts with the selection of data, analysis techniques to be used, and input parameters for the analyses, using the GUI. This set of choices is collectively referred to as the ‘configuration’. This selection is passed to the NeuroChaT backend, which then computes the specific analyses, and automatically plots and stores the graphical results to the storage disk. At the end of the analysis, a graphical table pops up showing the numerical results that the user can refer to for inferential analysis. These numerical outputs can be exported to an excel file, while graphical results are exported to a PDF file. NeuroChaT can store a specific configuration to be loaded again at a later date using the GUI.

***Batch-mode analysis.*** NeuroChaT facilitates batch mode processing by providing the unit and spatial information in an Excel list. Researchers often keep track of identified single units or units of interest using an Excel file; we facilitate this analysis using this list. The output graphics are, accordingly, all stored in the respective data folder. Units with speculated-upon similar properties, for example, head-directional firing, can be listed in one file for the convenience of post hoc inferential analysis of population data. The verification utility in the software can verify the information specified in the Excel list for batch processing, i.e. whether the specified path or files exist, or whether the cluster unit of interest belongs to the recording or is mistyped. This ensures the user does not waste time finding issues after running the analyses and knows ahead of about problematic specifications. As many of the NeuroChaT analyses are time consuming, this is a convenient way of eliminating common human errors and reduces time wasted.

***Nomenclature.*** NeuroChaT provides for better data management by standardising the nomenclature in its output data file. It creates a unique name for each unit of a recording session using the following format: unit_id = record_id+ ‘TT’+ tet_no+ ‘_SS_’+ unit_no + ‘_’+ eeg_file_ext where record_id = unique file or folder identifier for each recording session used to store and identify data, tet_no = electrode number where the unit is identified, unit_no = tag of the unit or the cluster number in spike-sorting, eeg_file_ext = filename or the extension used for naming an LFP data file. This approach brings efficiency to managing and scrutinising the outcome of data analyses. Current analyses in NeuroChaT can produce more than 50 graphical outputs for each unit with publication-quality images. Storing them in one file creates the initial layer of output data management. These output files are stored in the respective data folder, so they can be easily traced. The unique name ‘unit_id.pdf’ of the unit information is essential when working with many such units from the same study; otherwise, keeping track of the output graphics would be overwhelming in terms of the number of graphics files and the amount of disk space they would require.

***Converting data to a widely-accessible format.*** The proprietary format data are converted into HDF5 and are accessible through the HDF5 file viewers ( www.hdfgroup.org/), once they go through NeuroChaT. Every time NeuroChaT analyses a unit, it stores the analysed data in the HDF5 file as a group that has been named following the NeuroChaT convention described above. There is always one HDF5 file for one recording session but different groups for each recorded unit. The recorded data are stored following the specification as in the NWB format ^20^. NeuroChaT also has a utility that converts the unit data from a vendor format to HDF5 format using a data specification list like the one used for the batch-mode processing. Additionally, the NeuroChaT input output module for HDF5 through Nhdf enables writing data and attributes to any of its paths or data without rewriting the entire file which was a major limitation in the NWB API.

***Utility for graphics management.*** Given that many units are recorded over time, the number of pdf or ps output files grows linearly. The PDF management utility in NeuroChaT facilitates merging the output files of interesting units into one file or moving them to a folder to group them together. The utility can be used either by providing a list of units or by manually choosing the files using an interactive window. At the end of each execution, NeuroChaT provides a list of pdf files where the graphical outputs for each analysed unit are stored. Users can export this list from the GUI utility menu and can use the list for merging or accumulating them into one folder. Thus, NeuroChaT also bridges the gap of tracing, by using unique nomenclature and managing hundreds of graphical outputs in a logical approach.

## Use cases

### Assessment and validation of individual neurons

**Figure 6.**
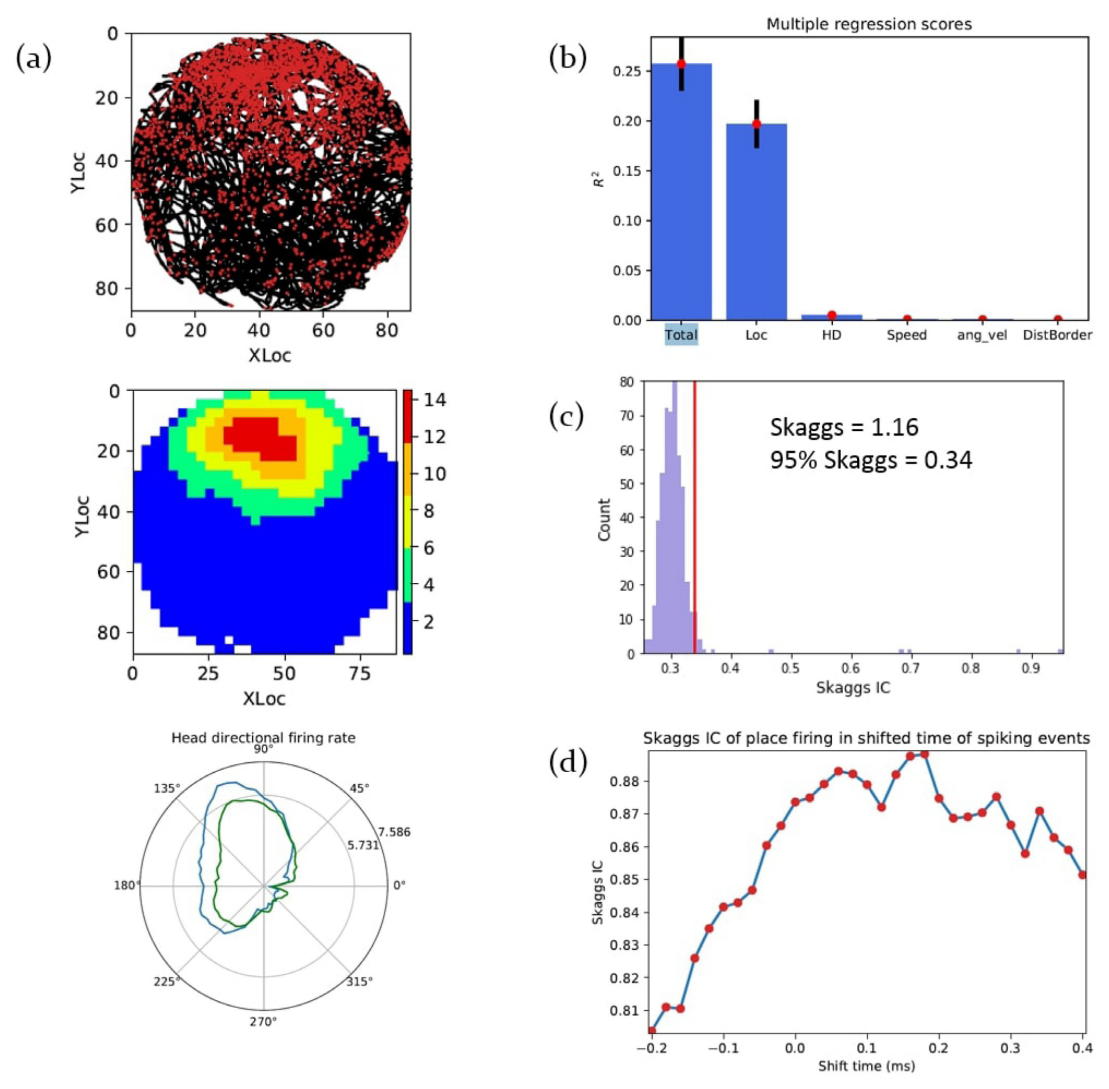
Identifying and verifying a place cell using analyses in NeuroChaT recorded in rat anterior thalamus ^7^. ( **a**) Top- the scatter plot of spiking-activity showing the path of the animal (black line) and the location in the arena where spiking activity occurred (red dots); Middle- the firing rate map of the unit showing the patch of locational receptive field. These two plots provide the initial screening of the place unit. Bottom- the firing rate of the unit with respect to the head-direction of the animal. The blue line shows the true rate and the green line is the predicted rate as described by Cacucci *et al.* ^24^. Both lines are very similar, implying that there is a sampling bias for the head-direction and, therefore, the tuned rate towards nearly north direction is not representative of the head-directional unit. ( **b**) Multiple regression analysis shows that location contributes to most of the variation in firing rate, confirming that the location is the main contributing factor and, in this case, the only factor to contribute to the spiking activity. ( **c**) The distribution of Skaggs in randomly shuffled spike time (no. of shuffles = 500). The 95th percentile (0.34) of the distribution is much lower than the Skaggs of the original activity of the unit (1.16), so the place cell activity is not random. ( **d**) The systematic changes in Skaggs information content (IC) as spiking timing is shifted by –0.2 s to 0.4 s in steps of 25 ms.

In 7, 21, we reported the presence of spatially-responsive neurons in the rat anterior and rostral thalamic nuclei. Consider one of the place cells as shown in Figure 6 ^22^. The top and middle row show where in the environment one such unit becomes active (spike-plot) and the firing rate map of the unit with respect to the 2D location of the animal. The patch of high firing zone implies that the unit is responsive to the location of the animal. This patch of firing could result from three different factors:

1. The unit may fire with respect to that part of the border, as in boundary vector cells ^23^.
2. The animal might face the north wall of the environment while approaching that area and a head directional unit may appear as a place cell, because of constraints on the trajectory of movement, and therefore of the sampling of unit activity ( Figure 6; bottom).
3. The unit might also be a head direction-by-place cell, where the unit fires in a certain location of the environment only when it is heading towards a particular direction ^24^.

We can use multiple built-in analysis methods in NeuroChaT to assess and confirm whether the unit is a place cell as mentioned below:

1. Multiple regression analysis models the instantaneous firing rate of the unit by a linear combination of the environmental variables under consideration ^25^ and provides the relative contribution of each factor on the firing property of the unit. As the firing rates are idiosyncratic for place and head-directional cells, the variable values were replaced by the corresponding average firing rate maps.
2. Assuming the null hypothesis that the observation of a place cell is a matter of chance, we can do shuffling analysis. In this technique, neuronal spikes are randomly shuffled and the specificity index (Skaggs Information Content) ^18^ for each such artificial unit is calculated. The specificity index of the unit is tested whether it is significantly larger than the mean information content in a population of shuffled simulated units firing randomly with respect to the location.
3. Finally, we can perform the time-shift analysis and observe whether the unit follows a gradual change in information content with respect to the time-shift, implying that the firing rate is not random, and there is a consistent and graded, or a systematic location-related variation.

The multiple regression for this unit ( Figure 6b) shows that the variation in spiking activity is primarily due to the location of the animal and is not merely due to other factors. Skaggs distribution shows that the information calculated from the original spiking activity is greater than 95 *th* percentile of the distribution in random spiking, implying that the specificity to locational firing is significantly larger than the randomly-correlated units and, therefore, the null hypothesis of observing the locational firing of the unit by chance is not true. The time shift analysis, although not very smooth, still shows that there is a graded change in the information content, marked by the parabolic change in information content as the timing of the unit-activity is gradually shifted by –200 ms to 400 ms, which further implies that the effect of location on the firing rate is systematic rather than random.

### Assessment and validation of a population of neurons

**Figure 7.**
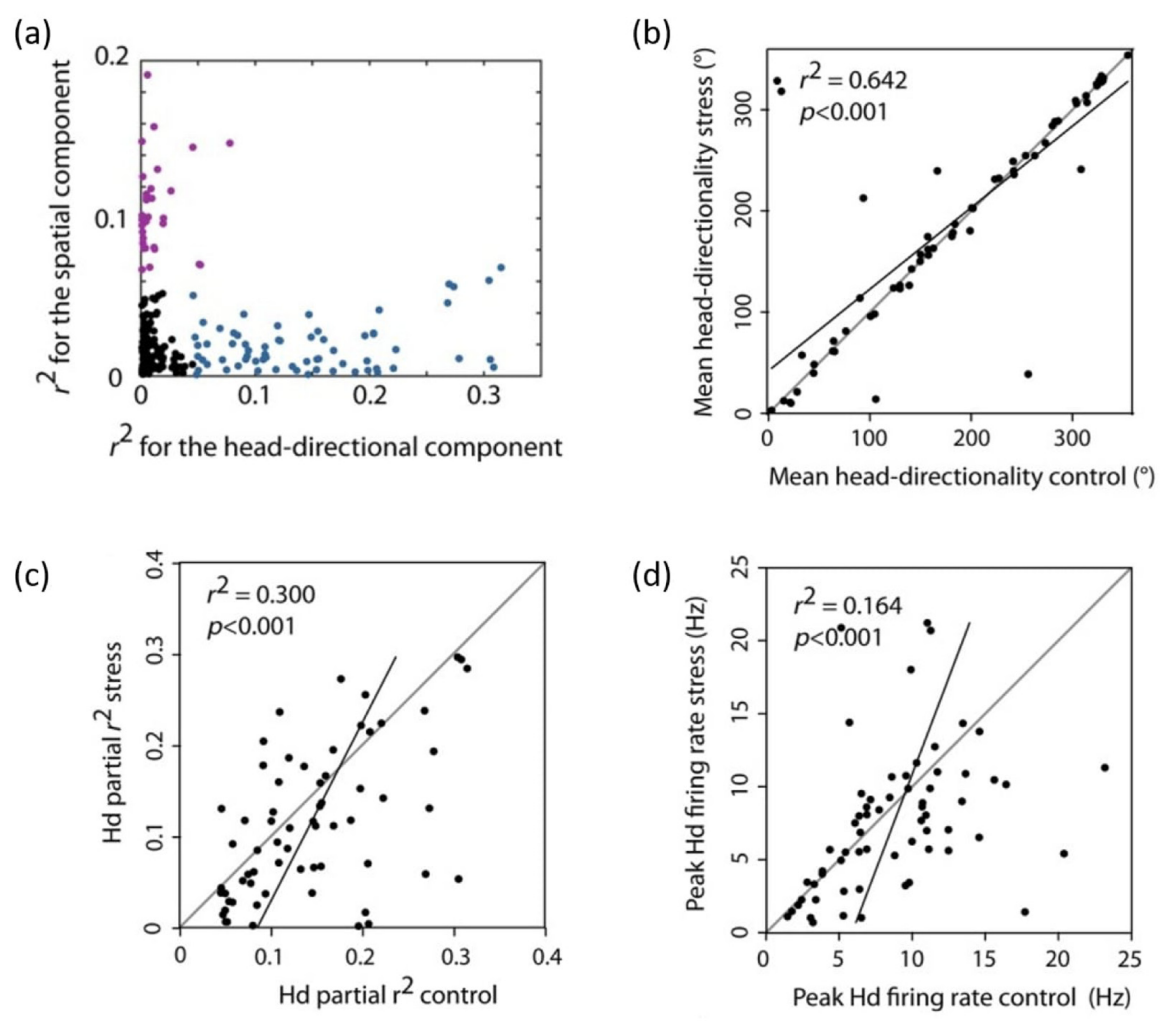
Study of the effect of photic stress on postsubicular head-directional cells (adapted from 26). NeuroChaT output parameters were used to accomplish the study. **a**) Identification of head-directional cells of recorded postsubicular cells. Correlation coefficients from multiple linear regression analysis and subsequent cluster-analysis revealed spatial (purple) and head-directional (blue) cells. The head-directional cells are not changing their preferred direction of firing, as shown by the high correlation between the mean directions of the neurons before and after stress induction ( **b**), but the correlation coefficient representing the variability of the firing rates due to head-direction ( **c**) and the peak firing rates ( **d**) changes. This implies that information processing is disrupted due to stress experienced by the rats.

The analysis outcome in NeuroChaT has been used to assess the effect of stress induced by high-intensity light exposure to rats on its spatial information processing system, particularly on units that represent the head-directional information in postsubiculum of the hippocampal formation (HF) ^26^. Following the initial cell selection, 230 units were analysed using NeuroChaT ^22^. The units with dominant head-directional firing were identified using supervised k-means clustering of the distribution of multiple-regression coefficients ( Figure 7a) for location and head-direction. We did not find significant correlations for border angular head-velocity and running speed of the animal. Sixty-five head-directional (HD) cells were identified to be in a distinct cluster, representing higher correlation to direction. Several of NeuroChaT’s numerical outputs such as the preferred firing direction of the HD cells and the peak firing rate were used for characterising the units and comparing the changes in these characteristics due to stress. Each unit remained a stable predictor of direction in both the conditions as the preferred head-directionality of units remained unaffected ( Figure 7b; Pearson’s *r* ^2^ = 0.64, *p <* 0.001) and the accuracy of directional representation, as measured by the half-width of the directional tuning curve was unaltered (Pearson’s *r* ^2^ = 0.715, *p <* 0.001). Head-directional partial *r* values and peak head-directional firing rate variables showed a significant decrease in value ( Figure 7c and d; mean head-directional partial *r* value *Z*65 = –3.029, *p* = 0.002, peak head-directional rate *Z*65 = –2.109, *p* = 0.035). A number of other aspects were also studied, such as assessing whether the photic stress influences a specific sub-population of head-direction cells, see Figure 7 (adapted from 26).

### Assessment of rhythmic properties of a neuron

Spike-train dynamics and the nature of the interaction with simultaneously recorded LFP provides vital information to understand the neuronal networks and the dynamics of individual neural components across different brain areas ^27, 28^. Analysis of this sort can be important particularly for assessing the mechanism of spatial computation as it is hypothesised that there is a spatial information packaging by theta rhythms ^29^. The cortical head-directional cells are segregated in time by alternating theta cycles according to their directional preference ^27^, hippocampal place cells show location specific phase-segregation reflecting the distance representation by time-compression, which is also dependent on speed of the animal ^30^, and separate theta cycles segregate distinct environmental representations and the changes in context, i.e. location of reward ^28^. Analysis of theta-modulated units, theta-skipping units, and units to LFP phase synchrony are widely used in this regard. In NeuroChaT, we assess them using the following analyses:

1. The distribution of ISI, and the relationship of the interval before vs interval after.
2. The autocorrelation histogram of ISI, which exfoliates the rhythmic pattern merely observed for the ISI itself.
3. The distribution of LFP-phases at the time of the unit activity ^31^, the phase-locking value (PLV) ^32^ and the spike-field coherence (SFC) ^33^ at different frequencies.

**Figure 8.**
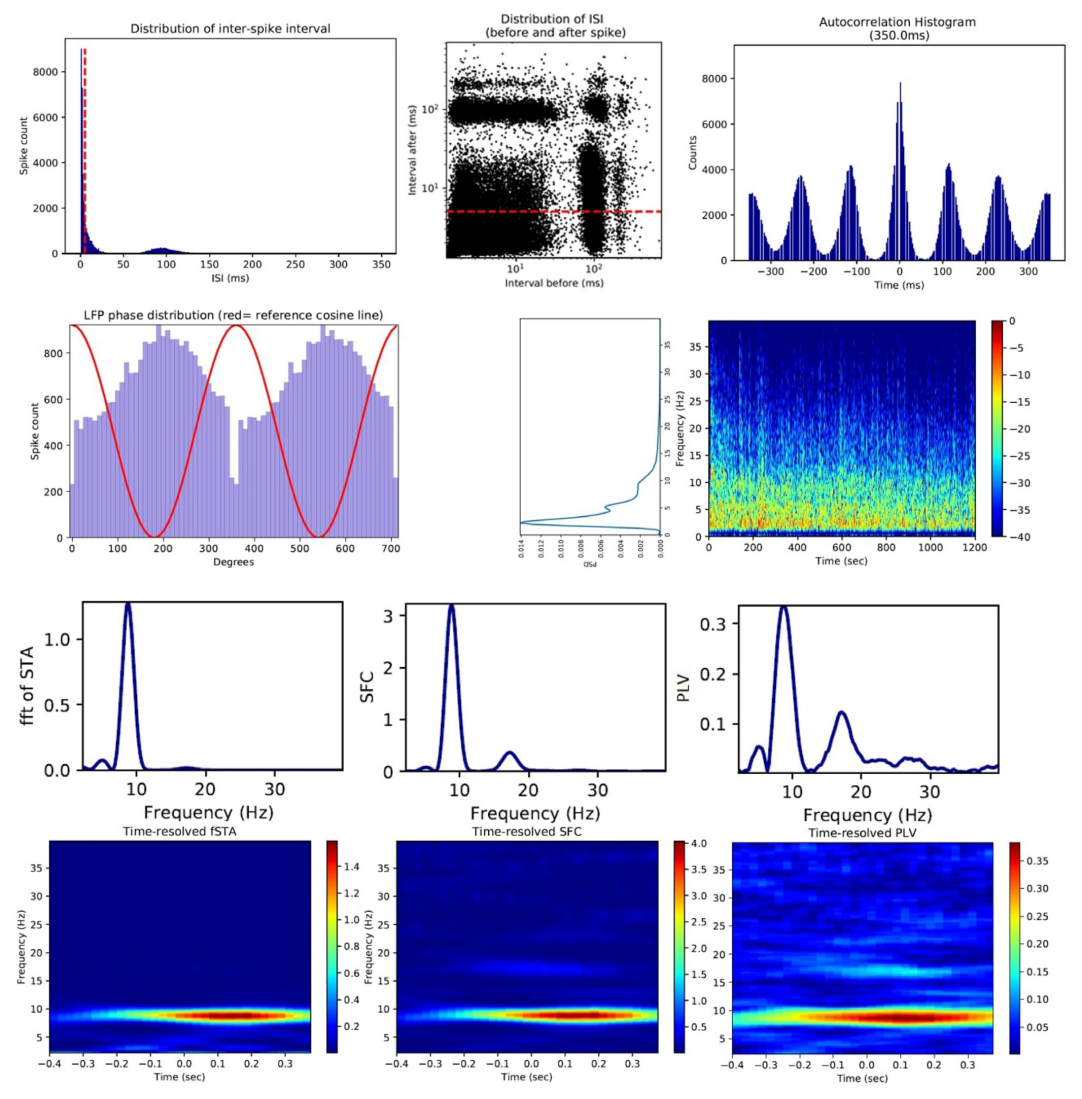
Upper row: left- inter-spike interval (ISI) distribution revealing that this unit has high burst propensity and is theta-rhythmic; middle-ISI before vs after discloses the characteristic patches at around 125 ms indicating high replication of such ISI events; right- autocorrelation of ISI histogram amplifies the rhythmic effect. Middle row: left- distribution of spike phases with underlying local field potential (LFP) signal; right- LFP power spectrum showing that there is a presence of weak theta-rhythm in the LFP. Lower row: although the LFP theta is small, the frequency spectrum of spike-triggered average (STA; left), the spike-field coherence (SFC; middle) and phase-locking value (PLV; right) all display strong locking to theta signal, verifying the locking as seen in phase distribution. Bottom row: the time-resolved fast Fourier transform (FFT) of STA (left), SFC (middle), and PLV (right) show that the peak locking does not occur simultaneously and has a lag from the time of spike-onset. It may imply that the LFP phase is encoding the spiking event instead of momentary representation or prediction of the spikes. The lag time for peak metric is 125 ms, which may also imply that the spiking event is represented in the next theta cycle instead of the synchronous one.

A unit with clear rhythmicity in firing activity represented by a higher count of ISI at around 125 ms is shown in the upper row of Figure 8 ^22^. This unit was co-recorded with head-directional cells in the electrophysiology study of thalamic nuclei ^7, 21^. The scatter plot of ISI before and after shows distinct patches implying the replication of ISI at those values (roughly 125 ms). The autocorrelation histogram unfolds the rhythmicity more prominently. As the replication occurs at around 8 Hz or in the Theta-rhythmic band, this unit is called a theta-modulated cell. Further analysis of this unit provides its descriptive characteristics. The spike to LFP phase distribution shows that there is a higher count of phases at around 195°. Although the delta band signal dominates the underlying LFP, the unit is still strongly locked to the theta-band as can be seen from the high PLV and SFC at around 10 Hz. The time-resolved PLV and SFC analysis of the unit provides further insight into the temporal nature of the locking. As the bottom row of Figure 8 shows, the locking is maximal at around 10 Hz throughout the entire window, but it evolves after the spiking event and maximises at a lag of roughly 125 ms, implying that the theta phase encodes the spiking event. One interpretation of the 125 ms lag for maximal locking is that the spiking event is encoded in the next cycle of the theta wave.

## Discussion

We developed the NeuroChaT toolbox to facilitate and standardise the analysis of neuronal spike trains and their relationship to behaviour and to simultaneously recorded LFP signals. NeuroChaT is hosted in a GitHub repository ( https://github.com/shanemomara/omaraneurolab). We provide a simple graphical interface and an easy-to-use API for using the corresponding analysis techniques and managing data. We hope that providing a simple, easy to use software package will facilitate the adoption of *in vivo* recording techniques. We hope that NeuroChaT, by assembling standard analyses techniques in one place along with a standard workflow will facilitate the adoption of standardised analyses for behavioural neurophysiology, and facilitate open data sharing and collaboration between laboratories. The simple GUI is designed for researchers without programming knowledge, while the versatile design in API provides an opportunity for neuroscientists with programming expertise to use the platform as a starting tool for extending their analytic capabilities. The built-in collection of analyses methods will allow them to quickly scan and infer the characteristics of the recorded neurons and refine their experimental protocols. The examples both here and in the project documentation depict how NeuroChaT can be used to build a custom analysis portfolio for characterising single units and population of neurons.

Some commercial and open-source toolboxes, such as Neo ^34^, support conversion of electrophysiology data from several copyrighted formats (i.e. Axona, Blackrock, Plexon, NeuroExplorer etc.) to HDF5 format. NeuroChaT currently supports Axona and NeuraLynx formats. Integrating other data formats will be useful to provide for the analytic need of scientists using recording systems from a wide range of vendors. Currently, NeuroChaT supports analyses that pertain to assessing the dynamics of spatial correlates of neuronal responses. Analysis of stimulus-response dynamics is also widely studied in neurophysiology. Extensive development of event-related analysis using both the LFP and single-unit data will potentially open the door for wide-spread reception among neurophysiologists. An effort to integrate or to interface popular automated spike sorting algorithms or toolboxes can also be undertaken. Although there are frameworks for LFP-LFP ^35^ and point-process causality analysis between spike-trains ^36^, as far as we are aware, there is no such framework for studying the causal relations between the spike-train of a unit and simultaneously recorded LFP signals. Future work will pursue this aspect of analysis as well. Owing to the rise of big data in neurophysiology and envisioning the use of cloud computing ^37^, future developments of NeuroChaT can target a cloud-native version to support distributed computing and work with algorithms to support such technologies.

## Data availability

### Underlying data

Open Science Framework: NeuroChaT: Neuron Characterization Toolbox. DOI: https://doi.org/10.17605/OSF.IO/642YH ^22^.

This project contains the following underlying data:

- Example Place cell: 040513_1.hdf5 (Assessment and validation of individual neurons - neuronal data to reproduce Figure 6. These data were recorded by Maciej Jankowski ^7^.)
- Data from Passecker et al 2018.xlsx (Assessment and validation of a population of neurons - spreadsheet data containing the numerical output from NeuroChaT used to create Figure 7 (adapted from 26). These data were recorded by Johannes Passecker ^26^).
- Example theta modulated cell_conjunctive speed cell: 112512_1.hdf5 (Assessment of rhythmic properties of a neuron - neuronal data to reproduce Figure 8. These data were recorded by Maciej Jankowski ^7, 21^).

### Extended data

Open Science Framework: NeuroChaT: Neuron Characterization Toolbox. DOI: https://doi.org/10.17605/OSF.IO/642YH ^22^

This project contains the following extended data:

- Example Border cell: 040114_C3.hdf5 (recorded by Paul Wynne).
- Example Gradient cell_conjunctive angular head velocity and speed cell: 052214_C1.hdf5 (recorded by Pual Wynne).
- Example Grid cell: 120213_26.hdf5 (recorded by Maciej Jankowski).
- Example Head Directional cell: 120412_1.hdf5 (recorded by Maciej Jankowski).

Data are available under the terms of the Creative Commons Zero "No rights reserved" data waiver (CC0 1.0 Public domain dedication).

## Software availability

- An executable version of NeuroChaT for non-coder Windows users is available from: https://github.com/shanemomara/omaraneurolab/releases/download/v1.1.0/NeuroChaT.exe
- Source code available from: https://github.com/shanemomara/omaraneurolab/tree/master/NeuroChaT
- Archived source code at time of publication: https://doi.org/10.5281/zenodo.3543732
- License: GNU General Public License version 3
