# Supplementary material for: NeuroChaT: A toolbox to analyse the dynamics of neuronal encoding in freely-behaving rodents in vivo
Source: Wellcome Open Res. 2019 Dec 9;4:196. [Version 1] doi: 10.12688/wellcomeopenres.15533.1 (PMC7001759; doi:10.12688/wellcomeopenres.15533.1)
Supplement: Peer review history

# Peer review: Reviewer response for version 1

**DOI:** 10.21956/wellcomeopenres.17003.r37544

**Published:** 2020-02-04

**Authors:** Benjamin J. Clark, Ryan E. Harvey

**Affiliations:** 1 Department of Psychology, University of New Mexico, Albuquerque, NM, USA

Islam et al. describe the functionality, architecture, and potential impact of their software which is designed to analyze co-recorded in-vivo electrophysiology and behavioral data. Further, the authors explore how the widespread adoption of this software may benefit the field at large via the adoption of standardize methodologies and open source data formats. Thus, the current project represents an important step toward the availability of open source tools. We agree that this software will aid in the standardization of methodologies and data formats in the field of in-vivo electrophysiology. While the authors have provided a useful toolbox, we suggest a number of  improvements to the user friendliness and transparency of the software which we feel is ultimately in line with the author’s goals for this project. Our major comments are listed below.

1. Introduction - first paragraph – the authors should also make note of recent developments in egocentric coding [e.g., Hinman et al., (2019) ^1^; LaChance et al., (2019) ^2^].
2. Methods - third paragraph under heading “Analysis of methods”: the authors should provide a definition and citation of “gradient cell”. 
3. Methods - third paragraph under heading “Analysis of methods”: Can the authors make note of the number of bins required to meet the threshold for a place field definition? How many place fields can be defined for each cell?
4. Related to our comments above, more detail is needed in describing methods for each category of analysis. For example, there are several methods to calculate “theta-modulated cell index,” and some methods may not be appropriate for low firing units (Climer et al., 2015 ^3^). Please make explicit which specific methods are being employed for each measure.
5. Using the software’s .exe form, while we were able to use the available test files, we were unable to successfully conduct any analyses using our own Neuralynx data. Some issue exists which made the program crash upon initiating each analysis. As it stands, we were unable to fully test this software.  
6. A list of acceptable spike-sorted data formats should be listed. To be more user friendly, the integration of multiple spike sorted data formats should be considered for integration such as results from popular methods: Phy, MClust, Mountainsort, Offline Sorter, etc. Otherwise, the software in its current form will require users to write unit identities back into original Neuralynx and Axona formats.  
7. We suggest that the authors make use of perceptually uniform colormaps which is friendly to common forms of colorblindness. Further, does the software allow adjustment of colormap resolution? For example, in figure 6a row 2, the colormap is down-sampled to a handful of color levels resulting in a loss in the available firing rate resolution (e.g., 2Hz is identical to 0Hz). 

We confirm that we have read this submission and believe that we have an appropriate level of expertise to confirm that it is of an acceptable scientific standard.

## References

[1] : Neuronal representation of environmental boundaries in egocentric coordinates. *Nature Communications*.2019;10(1) : 10.1038/s41467-019-10722-y 10.1038/s41467-019-10722-y PMC659116831235693

[2] : A sense of space in postrhinal cortex. *Science*.2019;365(6449) : 10.1126/science.aax4192 10.1126/science.aax4192 31296737PMC7063980

[3] : Examination of rhythmicity of extracellularly recorded neurons in the entorhinal cortex. *Hippocampus*.2015;25(4) : 10.1002/hipo.22383 460-73 10.1002/hipo.22383 25331248PMC4457388

# Peer review: Reviewer response for version 1

**DOI:** 10.21956/wellcomeopenres.17003.r37546

**Published:** 2020-02-03

**Authors:** Anna S. Mitchell, Brook A. L. Perry, Eleonora Lomi

**Affiliations:** 1 Department of Experimental Psychology, Oxford University, Oxford, UK

NeuroChaT is a toolbox written in Python that allows a number of measures to be computed based on an animal’s neurophysiological data and simultaneously recorded spatial information. Its’ application is particularly useful for the analysis of spatially-specific neural activty. The software has been written to use output files generated in Axona and NeuraLynx electrophysiology recording systems, or from other proprietary electrophysiology systems with conversion to the HDF5 file extension format. We agree with the comments made by Poucet and Jacob (2019), especially the suggestion of a short manual that provides formulas to indicate how some of the measures were computed. We have additional comments based on our experience using NeuroChaT.

 For this review, we were able to trial the executable GUI version of the NeuroChaT analysis software for Windows 10. Thus, our comments may not apply to the NeuroChaT toolbox if it is generated from the source code. We analysed data acquired using the Axona dacqUSB data acquisition system and analysed using the TINT cluster cutting program. The electrophysiology recording session was conducted in our lab with a rat running around in a square open field arena. To get started with the analysis, the GUI requires the spike times and waveforms information for a tetrode (spike .n  file) and a .txt file containing the tracker position data. In order to generate this file, TINT is used to produce a “.txt” file from the “.pos file”, which contains a vector of X - Y spatial coordinates of the rat during the recording session. Although the dialogue box indicated that a spike .n file and/or a spatial .txt file could be used, we were unable to get the GUI to work using only a spike .n file, even if the output analyses that we selected did not require any positional  information. Nonetheless, the instructions were easy to follow and NeuroChaT created a .pdf file and a numerical output file of our data. 

 Our comments relate to clarification of the .pdf file and the numerical output file.

 Reassuringly, our figures previously generated from custom written code and functions in Matlab matched the equivalent output files of NeuroChaT. In addition, NeuroChaT provided several additional figures that we think are very helpful. For example, the figure showing changes in head direction tuning curves as a function of time and displaying changes in tuning parameters minute by minute. This analysis is useful to visualize preferred firing direction fluctuations around the mean direction, which is not captured by commonly used measures of linear drift. Although there may be sampling bias issues due to the time the rat spends looking toward the preferred firing direction at different time periods, it would be helpful to have some values for these plots, in order to characterise preferred firing direction fluctuations. We suggest a measure of instantaneous drift rate in degrees/minute. This defines the direction and the extent of drift, computed by looking at differences in preferred firing direction between adjacent time periods.

 The decomposed clockwise and counter-clockwise tuning functions plot shows the extent of angular separation which enables cells to anticipate the actual head direction. We may suggest to add to this plot a legend showing which of the two clockwise and counter-clockwise curves are represented by the two colours (red and blue). Moreover, a measure of angular separation between the two decomposed functions would be useful, in order to be correlated with the anticipatory time interval analysis. 

 The program also includes a different way to quantify anticipatory firing of a head direction cell. This is useful, as the standard anticipatory time interval measure commonly used in spatial navigation papers has some drawbacks. First of all, this measure is affected by the smaller number of observations at high angular head velocity values. Secondly, it is difficult to compare this measure across brain regions, as researchers have often used different head direction and angular head velocity bins. The alternative measure implemented by NeuroChaT is based on the optimal time shift value that is required to optimise tuning curve parameters. The relation between optimal time shift and peak firing rate/ Skaggs information content is plotted. It would be useful to generate figures for the relation between tuning width and shift value. 

 One of the strengths of this program is that it is straightforward and easy to use. It would be a good idea to implement also a GUI version that allows to upload more than one trial at a time, for example to compute a mean spectrogram for the entire session. Similarly, the program conveniently and quickly writes the numerical output associated to each plot in an Excel file. We noticed that these files, at least in our hands, were overwriting each other as we conducted subsequent analyses of different units within the same folder so it might be useful if new file names could be generated for each analysis, possibly with the filename of the spike .n file. 

 The local field potential (LFP) phase – coherence analysis is very detailed, and provides plots to visualise the extent of phase locking (Rayleigh vector) and preferred phase of each cell. In the polar histogram named “LFP phase distribution plot”, we suggest that the red line detailing the preferred phase of each cell should be proportional to the Rayleigh vector length (the theta modulation depth of that cell). This is because for irregularly firing cells, the mean phase is not a representative measure, as cells do not increase firing on any specific theta phase. Moreover, to avoid binning the phase data, a polar scatter plot could be implemented alongside the polar histograms in which each dot represents the phase of the underlying oscillations at which each spike is emitted. Finally, we think having the option to run the spike-LFP and LFP related functions independent of the spatial data.

 For the future, it would be helpful for our lab, to broaden the type of accepted file formats at least to be more compatible with Matlab or Plexon. This would be of great utility to analyse monkey electrophysiology data and quantify spatial firing (e.g. in head direction cells) across different species.  

 Overall, NeuroChat provides a standardized approach to the analysis of spatial-specific neural data that allows interpretation and comparison across labs, making results more reliable, and reproducible.

We confirm that we have read this submission and believe that we have an appropriate level of expertise to confirm that it is of an acceptable scientific standard.

# Peer review: Reviewer response for version 1

**DOI:** 10.21956/wellcomeopenres.17003.r37320

**Published:** 2019-12-19

**Authors:** Bruno Poucet, Pierre-Yves Jacob

**Affiliations:** 1 CNRS, LNC UMR 7291, Aix-Marseille University, Marseille, France

The authors provide a toolbox that contains most of, if not all, useful analyses to characterize the main firing properties of neurons that code space in one way or another (place cells, head direction cell, grid cells, etc.). As mentioned in the introduction of their paper, most research groups in the field have developed their own custom written code for data analysis, but the lack of methodological standardization prevents direct comparisons between the data acquired here and there. There is therefore a clear advantage to having this toolkit, especially for newcomers to the field.

 The toolbox as described in the article appears to be well designed, user-friendly and comprehensive. Unfortunately, we tried to run the executable version of the software on data files recorded with Axona or NeuraLynx systems and could not go beyond the main menu. The problem apparently stems from the heterogeneity of the data format, not only between systems but also within systems. This is probably where the toolkit can be improved, by trying to make it compatible, in a user-friendly way, with most of the formats used for recording and storing behavioral (e.g., positional, user-entered events) and neural (single unit and LFPs) data.

 Among other possible improvements that could be considered, it would be useful to have a short manual explaining briefly the analyses and possible settings and providing the formulas (e.g., how spatial coherence, Skaggs information, Rayleigh vector length, theta index, etc. are computed)

 Understanding neural substrates for spatial processing is a rapidly evolving domain. Although the toolkit is capable of assessing the spatial firing characteristics of the "historical" classes of spatial neurons (e.g., place cells, head direction cell, etc.), upgrading of the toolkit will need to take into account the recent discovery of neurons that may have more complex correlates (e.g., goal-distance cells: Spiers et al., Hippocampus, 2018 ^1^; goal-direction cells: Sarel et al., Science, 2017 ^2^; bidirectional and multidirectional cells: Jacob et al., Nat Neuro, 2017 ^3^ and Olson et al., Nat Neuro, 2017 ^4^; egocentric boundary cells: Hinman et al., Nat Comm, 2019 ^5^). For example, recent advances have emphasized the importance of goal location or environmental geometry. The ability to process data by entering areas of spatial interest will be a great improvement.

We confirm that we have read this submission and believe that we have an appropriate level of expertise to confirm that it is of an acceptable scientific standard.

## References

[1] : Hippocampal CA1 activity correlated with the distance to the goal and navigation performance. *Hippocampus*.28(9) : 10.1002/hipo.22813 644-658 10.1002/hipo.22813 29149774PMC6282985

[2] : Vectorial representation of spatial goals in the hippocampus of bats. *Science*.2017;355(6321) : 10.1126/science.aak9589 176-180 10.1126/science.aak9589 28082589

[3] : An independent, landmark-dominated head-direction signal in dysgranular retrosplenial cortex. *Nat Neurosci*.20(2) : 10.1038/nn.4465 173-175 10.1038/nn.4465 27991898PMC5274535

[4] : Subiculum neurons map the current axis of travel. *Nat Neurosci*.20(2) : 10.1038/nn.4464 170-172 10.1038/nn.4464 27991899

[5] : Neuronal representation of environmental boundaries in egocentric coordinates. *Nature Communications*.2019;10(1) : 10.1038/s41467-019-10722-y 10.1038/s41467-019-10722-y PMC659116831235693
